# Field Evaluation of Personal Sampling Methods for Multiple Bioaerosols

**DOI:** 10.1371/journal.pone.0120308

**Published:** 2015-03-23

**Authors:** Chi-Hsun Wang, Bean T. Chen, Bor-Cheng Han, Andrew Chi-Yeu Liu, Po-Chen Hung, Chih-Yong Chen, Hsing Jasmine Chao

**Affiliations:** 1 School of Public Health, Taipei Medical University, Taipei, Taiwan; 2 Health Effects Laboratory Division, National Institute for Occupational Safety and Health, Morgantown, West Virginia, United States of America; 3 Institute of Labor, Occupational Safety and Health, Ministry of Labor, New Taipei City, Taiwan; University Paris South, FRANCE

## Abstract

Ambient bioaerosols are ubiquitous in the daily environment and can affect health in various ways. However, few studies have been conducted to comprehensively evaluate personal bioaerosol exposure in occupational and indoor environments because of the complex composition of bioaerosols and the lack of standardized sampling/analysis methods. We conducted a study to determine the most efficient collection/analysis method for the personal exposure assessment of multiple bioaerosols. The sampling efficiencies of three filters and four samplers were compared. According to our results, polycarbonate (PC) filters had the highest relative efficiency, particularly for bacteria. Side-by-side sampling was conducted to evaluate the three filter samplers (with PC filters) and the NIOSH Personal Bioaerosol Cyclone Sampler. According to the results, the Button Aerosol Sampler and the IOM Inhalable Dust Sampler had the highest relative efficiencies for fungi and bacteria, followed by the NIOSH sampler. Personal sampling was performed in a pig farm to assess occupational bioaerosol exposure and to evaluate the sampling/analysis methods. The Button and IOM samplers yielded a similar performance for personal bioaerosol sampling at the pig farm. However, the Button sampler is more likely to be clogged at high airborne dust concentrations because of its higher flow rate (4 L/min). Therefore, the IOM sampler is a more appropriate choice for performing personal sampling in environments with high dust levels. In summary, the Button and IOM samplers with PC filters are efficient sampling/analysis methods for the personal exposure assessment of multiple bioaerosols.

## Introduction

Ambient bioaerosols are ubiquitous in the daily environment. Bioaerosols are airborne particles that are living or originate from living organisms, such as microorganisms and fragments, toxins, and metabolites from living beings [1]. Since the 1990s, epidemiological and toxicological studies have shown a close association between exposure to bioaerosols and many adverse health effects, such as infectious diseases, acute toxic effects, allergies, and cancer [1–3]. Therefore, exposure to bioaerosols is a crucial occupational and environmental health issue that warrants close attention.

The workers in industries, such as health care, agriculture, fishery, forestry, mining, construction, and day care are exposed to higher risks of biological hazards because of the work characteristics. Numerous studies have indicated that these workers have higher prevalence rates of respiratory diseases and airway inflammation [4–10]. However, few studies have been conducted to comprehensively evaluate personal bioaerosol exposure in occupational or indoor environments [11–13], mainly because of the complex composition of bioaerosols, and the lack of standardized sampling/analysis methods [1]. Without appropriate personal exposure assessment and standardized sampling/analysis methods, establishing dose-response relationships and relevant exposure guidelines are difficult.

Personal sampling is the ideal method for assessing personal bioaerosol exposure. An ideal personal bioaerosol sampler used in occupational environments should be light and sturdy, noninterfering with the daily routine, able to collect target bioaerosols, and high in physical and biological sampling efficiencies [14]. In addition, because the types and levels of bioaerosols vary widely with occupational environments, the sampling/analysis method should enable the collection of various bioaerosols, with long (e.g., 8 h) and short (e.g., 15 min) sampling periods for evaluating various conditions.

Relatively few studies have been conducted to evaluate the efficiencies of personal exposure assessment methods for multiple bioaerosols [15, 16]. Therefore, the major objective of this study was to determine the most efficient collection/analysis method for the personal exposure assessment of multiple bioaerosols in indoor and outdoor environments. Although the overall efficiency of a bioaerosol sampler is determined by the sampling inlet, particle removal, biological recovery, and assay efficiencies [17], the differentiation of these individual efficiencies was not the objective of this study. Instead, we focused on comparing the overall efficiencies of the sampling/analysis methods. In addition, we evaluated the performance of the selected method in an occupational environment with high biological exposure.

## Materials and Methods

### Experimental design

A literature review was conducted to search for studies in which the authors used personal samplers to monitor multiple bioaerosols. The following databases were searched for relevant articles: PubMed, Medline, Web of Science, Scopus, and Google Scholar. The search terms used were as follows: “bioaerosol,” “personal sampler,” and “occupational environment.” We selected collection/analysis methods for further testing based on the following determining criteria: the sampling/analysis efficiency, the ease of application in occupational environments, suitability for multiple bioaerosol monitoring, and the capability of long (e.g., 8 h) and short (e.g., 15 min) time sampling.

**Fig 1:**
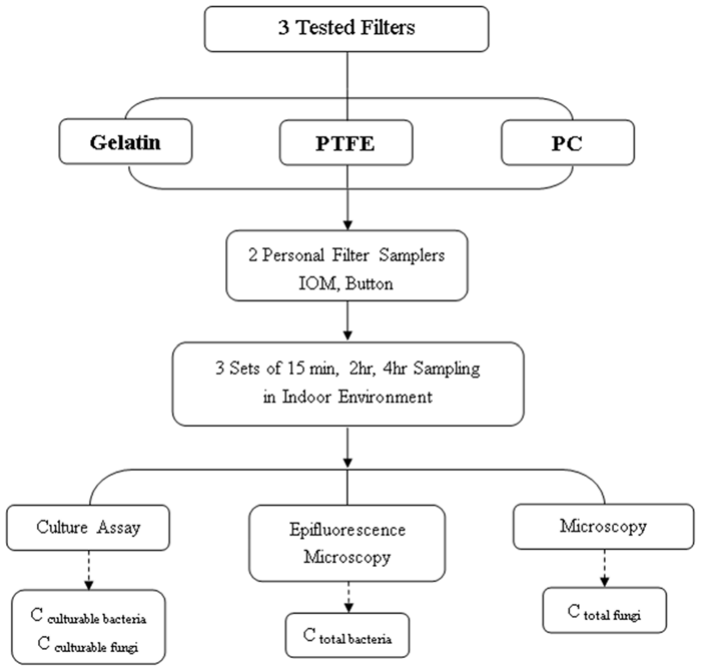
Flowchart for filter comparison.

**Fig 2:**
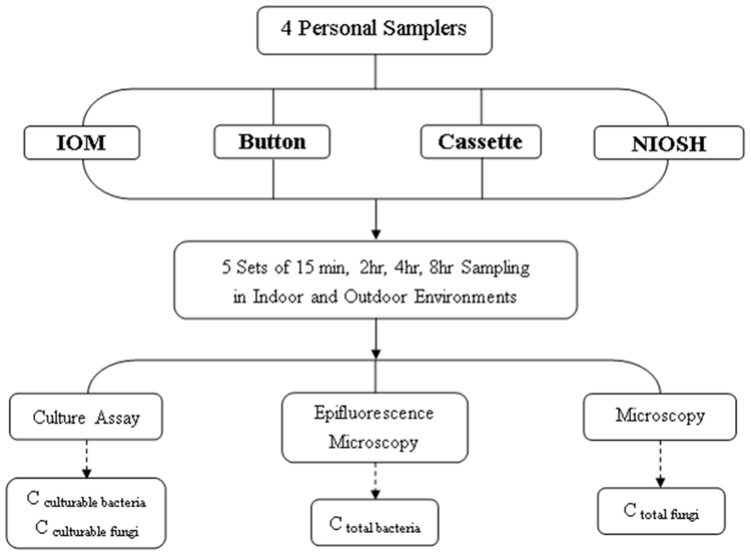
Flowchart for sampler comparison.

The relative efficiencies of the selected sampling/analysis methods were evaluated based on bioaerosol concentrations and biodiversity. The relative efficiencies of the commonly used filters were first compared in an indoor environment (Fig. 1). Afterward, the filter that obtained the highest bioaerosol ingredients in the analysis was used for the subsequent sampler comparison. The personal sampler comparisons were conducted in both indoor and outdoor environments (Fig. 2). The sampler that recovered higher bioaerosol concentrations and biodiversity was considered to have a higher relative efficiency. The performance of the selected samplers was further evaluated in a pig farm, an occupational environment with high biological exposure.

### Sampling methods

Based on literature review and the aforementioned determining criteria, we selected four personal samplers and three filters to evaluate their overall collection/analysis efficiencies [15,16,18–24]. The four tested personal samplers included the Button Aerosol Sampler, the IOM Inhalable Dust Sampler, the NIOSH Personal Bioaerosol Cyclone Sampler, and the 37-mm Filter Cassette sampler. Most of the selected personal samplers are based on the filtration principle, mainly because of the determining criteria. The capability of performing long and short time sampling precludes culture-based impactors. The majority of glass-made samplers are not appropriate to easily perform personal sampling in occupational environments. The three tested filters were gelatin filters (pore size 3 μm), polytetrafluoroethylene (PTFE) filters (pore size 1μm), and polycarbonate (PC) filters (pore size 0.8 μm), which have been commonly used and proven to have high recovery efficiencies for multiple bioaerosols [15,16,20,22–23,25]. The crucial characteristics of the four tested samplers are listed in Table 1. The Button Aerosol Sampler is a filter sampler with a porous curved surface sampling inlet made of stainless steel. The IOM Inhalable Dust Sampler is a conductive plastic sampling head that consists mainly of a two-part filter cassette. Both of these samplers use 25-mm filters as sampling media to collect inhalable airborne particles. The 37-mm cassette is composed of conductive plastic to collect total suspended dust by using filters. The NIOSH Cyclone Sampler [20,26] is a one-stage sampler that collects air through a brass cyclone attachment into a 1.5-mL conical micro-centrifuge tube with a screw cap (CV80–15, Biomate, Taipei, Taiwan) and uses a 37-mm closed-face filter cassette as a backup filter. All the samplers were operated at 4 L/min, except the IOM sampler, which was operated at 2 L/min, according to the recommendations of the vendors/inventors or the literature review.

**Table 1:** Characteristics of the tested personal bioaerosol samplers.

Air sampler	Description of operation					
	Sampling inlet	Airflow rate (L/min)	Inlet efficiency	Internal size selective capability	Collection medium	Bioaerosol measurement
**IOM sampler**	A protruding circular inlet of 15-mm diameter	2.0	Follow inhalable convention	No	25-mm filter	CFU^*a*^, Spore, Cell
**Button sampler**	A curved surface with numerous 381-μm orifices	4.0	Follow inhalable convention	No	25-mm filter	CFU, Spore, Cell
**Filter Cassette sampler**	A closed-face cartridge with a 4-mm inlet orifice	4.0	-	No	37-mm filter	CFU, Spore, Cell
**NIOSH sampler**	A circular sampling inlet with a 1.99-mm orifice	4.0	100% for particles up to 16-μm diameter^*b*^	Yes	1.5-mL micro-centrifuge tube (>1.5 μm fraction) and 37-mm filter (<1.5 μm fraction)	CFU, Spore, Cell

^*a*^CFU: colony forming unit

^*b*^Chen et al. 2004 [26]

### Sampling sites

To compare the relative efficiencies of the sampling/analysis methods, we selected a regional hospital (121° 33' 48"E, 25° 1' 36"N), which is an indoor environment, and a traditional market (121° 34' 39"E, 25° 2' 35"N), which is an outdoor environment, as the sampling sites because of the high levels of culturable fungi and bacteria. To evaluate the field performance of the optimal collection/analysis method, a pig farm located in Southern Taiwan (120° 15' 3"E, 22° 44' 18"N) was selected. The pig farm has an area of 2900m^2^ and holds approximately 1000 pigs. Institutional review board approval was not required because the study involved only environmental monitoring. Sampling permissions were granted by Taipei Medical University Hospital, the Yongchun Market Management Committee, and the owner of the pig farm. The field studies did not involve endangered or protected species.

### Sample collection

We first compared the relative collection/analysis efficiency of the three selected filters (gelatins, PTFE, and PC filters) in an indoor environment by using two types of filter samplers: the IOM and Button samplers. Six samplers (three IOM and three Button samplers coupled with one type of filter each) were collocated 30 cm apart on a rack approximately 1.5 m above the ground to implement side-by-side sampling. Samples were collected from the lobby of a regional hospital in October 2011. All the samplers were operated simultaneously to collect three sets of samples for each sampling time (15 min, 2 h, and 4 h). Various time intervals were selected to cover short and long sampling periods. Eight-hour sampling was not performed because the gelatin filters could not tolerate such a long sampling period. The sampling flowchart is summarized in Fig. 1.

Personal sampler comparisons were conducted in both indoor and outdoor environments in October 2011 (Fig. 2). The three tested filter samplers (i.e., Button, IOM, and Filter Cassette samplers) fitted with the most efficient filter (PC filter) and the NIOSH sampler were tested in the lobby of a regional hospital and in a traditional market. Four samplers were mounted side by side, and five sets of samples were collected for each sampling time (15 min, 2 h, 4 h, and 8 h) at both sampling locations. Various time intervals were selected to cover short and long sampling periods, corresponding to the sampling periods for the short-term exposure limit (STEL) and the 8-h time-weighted average (TWA) in the occupational settings.

After the optimal collection/analysis method was determined, it was assessed in a private pig farm in December 2011 for its performance in personal bioaerosol sampling in an occupational environment with high biological exposure. The daily duties of the pig farm employees included feeding pigs and cleaning sheds, which span periods that are representative of high bioaerosol exposure. Personal exposure assessments were conducted over 2 days by researchers wearing three types of samplers concurrently and following the workers during their working hours. The sampling periods consisted of a full day (n = 1) and two half days (n = 2), covering a complete work shift and activities with high biological exposures, respectively. During the full-day sampling, the worker fed pigs and swept sheds (without water) in the morning, as well as and in the afternoon (sampling time 177 min). For the two half days, the worker fed pigs and swept sheds (without water) in the morning (132 min) and fed pigs and washed sheds (with water) in the afternoon (sampling time 101 min). The sampling time corresponded with the actual working hours of the workers.

All the personal samplers were connected to personal pumps (AirChek XR5000, SKC Inc., PA, USA) and were operated at 4 L/min, except the IOM sampler, which was operated at 2 L/min. The samplers were calibrated before and after each sampling by using a primary standard calibrator (Bio Defender 510, Bios International Co., Butler, NJ, USA), and the average flow rate was used to calculate the corresponding bioaerosol concentrations. All the samplers were sterilized before sampling. The Button samplers, the metal parts of the IOM samplers, the NIOSH samplers, and the micro-centrifuge tubes were autoclaved. The Filter Cassette samplers were sterilized using ethylene oxide. All the other parts were cleansed with 70% isopropyl alcohol. After sampling, the collected filter samples were removed from the samplers immediately and placed in pyrogen-free tubes by applying an aseptic technique. All the samples were shipped to the laboratory immediately, stored at 4°C, and extracted within one week. The samples collected in the pig farm in Southern Taiwan were shipped to the laboratory at 4°C within 24 h. For quality control, 10% of each of the lab and field blanks of the tested filters were maintained. No contamination was observed.

During filter and sampler comparisons, the temperature, relative humidity, wind speed, and the number of people in the area during sampling were recorded. For the field evaluation, the temperature, relative humidity, the levels of CO_2_, CO, total volatile organic compounds (TVOC), and suspended particulates (PM_2.5_, PM_10_, and total suspended particulates), as well as the wind speed were recorded every 10 min by using direct reading instruments (KD AirBoxx Indoor Air Quality Monitor, KD Engineering, Blaine, WA, USA; Met One Instruments, Inc., Grants Pass, OR, USA; VelociCalc Plus Air Velocity Meters 8836A, TSI Inc, Shoreview, MN, USA). All the instruments were calibrated by the vendors before sampling.

### Sample analysis

The extraction/analysis methods were standardized in this study according to the vendor/inventor recommendations or the literature review. All the filters, including the backup filters of the NIOSH sampler, were soaked in 5 mL of an extraction fluid (0.01% Tween 80 in distilled water). The gelatin filters dissolved in the extraction fluid directly. The PC and PTFE filters in the extraction buffer were vortexed for 2 min, followed by ultrasonic agitation for 15 min. The tubes of the NIOSH sampler were rinsed with 4 mL of the extraction fluid (1 mL at a time, and vortexed for 20 to 30 s each time). The eluates were analyzed for culturable and total microbial counts. Because culture-based methods cannot detect nonviable and non-culturable cells and underestimate total bioaerosol concentrations, we analyzed the samples using both culture-based and non-culture-based methods (i.e., total bacteria and fungal spores) to reflect the various aspects of microbial characteristics.

For culturable microbes, aliquots (0.1 mL) of the eluates with different dilutions (1:1, 1:10, and 1:100) were plated on eosin methylene blue (EMB) agar and blood agar plates (BAP) for culturable bacteria and on malt extract agar (MEA) plates for culturable fungi. The bacterial culture plates were incubated at 35^°^C for 16 to 24 h and sent to the Department of Laboratory Medicine, Taipei Medical University Hospital, for identification. The identification was based on colony characteristics (e.g., morphology, shape, size, and hemolysis pattern), Gram staining, cell characteristics (e.g., shapes and arrangement), and biochemical reactions (e.g., the utilization of a nutrient source, as well as the metabolic products). Total concentrations of the culturable bacteria were calculated using colony counts on BAP, which could support the growth of most bacteria. The fungal plates were incubated at 25°C for 7 days before counting and identification. All the fungal colonies were counted and identified morphologically to the genus and/or species level by using a stereo microscope and a light microspore at 800× magnification. Fungal identification was performed by an experienced microbiologist, and the results were validated using standard monographs for culturable fungi [27–30]. The concentrations of culturable microbes are reported in colony-forming units per cubic meter of air (CFU/m^3^).

For the total bacterial count, 1 mL of the eluate was filtered through a 25-mm black PC filter (pore size 0.2 μm) in a filter holder by using a vacuum pump. The PC filter was then stained with 1 mL of acridine orange (0.1 mg/mL) for 10 min, drained of excess stain, and mounted on a glass slide with a cover slip [31]. An epifluorescence microscope (IX81 Motorized Inverted Microscope, Olympus, New Orleans, LA, USA) was used to analyze the total bacterial count at 400× magnification. Forty fields were counted per slide, with 10 equidistant fields of four diameters (45° apart). The total bacterial concentration, *C*_*total bac*._, was calculated as follows:

$$C_{totalbac.}[{\text{cells/m}}^{\text{3}}]=N\times \left(\pi R^{2}/A\right)\times \left(V_{1}/V_{2}\right)/{\text{Volume of air sampled [m}}^{\text{3}}\text{]}$$ (1)

where *N* is the average microbial count of 40 fields, *R* is the effective radius of the filter (12.5 mm), *A* is the area of the microscopic field (0.2376 mm^2^), *V*_*1*_ is the amount of extraction fluid used (5 mL for the filter samples or 4 mL for the NIOSH tubes), *V*_*2*_ is the eluate amount used for analysis (1 mL), and the *Volume of air sampled* is the flow rate of the sampler multiplied by the sampling time [31].

For the fungal spore count and identification, 1 mL of the eluate was centrifuged for 10 min, and then 0.9 mL of the supernatant fluid was removed. The remaining concentrated eluate was vortexed for 2 min, and half of it (0.05 mL) was dropped on a slide, dried, and stained using glycerin jelly with phenosafranin. The samples were analyzed under a microscope at 800× magnification. Fungal spores were identified morphologically by an experienced microbiologist by using standard fungal spore monographs [32–34]. The identified spores were categorized according to the classification of the American Academy of Allergy, Asthma & Immunology’s (AAAAI) Aeroallergen Network as Ascospores, Basidiospores, *Alternaria*, *Arthrinium*, *Aspergillus/Penicillium*, *Botrytis*, *Cercospora*, *Cladosporium*, *Curvularia*, *Drechslera/Helminthosporium*, *Epicoccum*, *Fusarium*, *Nigrospora*, *Oidium/Erysiphe*, *Periconia*, *Peronospora*, *Pithomyces*, *Polythrincium*, Rusts, Smuts, *Stemphylium*, *Tetraploa*, *Torula*, *Ulocladium*, and other fungi. We classified the unidentifiable spores and known spores not otherwise categorized as “other fungi.” The concentrations of total fungal spores are reported in spores per cubic meter of air (spores/m^3^).

### Data analysis

We used bioaerosol concentrations and biodiversity as indicators to evaluate the performance of the tested filters and samplers. Because consistent effects of sampling times (i.e., 15 min, 2 h, 4 h, and 8 h) on the bioaerosol concentrations were not observed, we combined all the samples for statistical analyses. Because the bioaerosol levels were not normally distributed, nonparametric statistical tests were applied. Concentrations recovered from the different filters or samplers were compared using the Friedman test, which is a non-parametric method similar to a one-way analysis of variance (ANOVA) with repeated measures. The method is used to examine the difference among several related samples (i.e., collocated samples with identical sampling intervals). If a statistically significant difference (*p* < 0.05) was detected using the Friedman test, the Wilcoxon signed-rank test was used as a post hoc test to identify the two filters or samplers with a significant difference. The number of microbial taxa recovered using different filters or samplers was counted as an index for biodiversity. All the statistical analyses were performed using SPSS 18 (SPSS Inc., Chicago, IL, USA).

## Results

### Relative efficiency evaluation of filters

During the sampling periods for filter evaluation, the environmental conditions were consistent at the sampling site. The number of people in the area ranged from 43 to 55; the temperature was 22.3 to 22.9°C; the relative humidity was 66.5% to 72.5%; and the wind speed was 0.09 to 0.15 m/s.

Table 2 shows the total bacterial and fungal concentrations obtained using the three types of filters and the two types of samplers (n = 9 for each combination with a total n = 54). For the culturable and total bacteria, statistically significant differences were observed among the three types of filters (*p* = 0.007 and 0.0119 for the Button and IOM samplers, respectively). The highest bacterial concentrations were recovered from the PC filters. According to the post hoc tests, the culturable bacterial levels differed significantly between the PC and gelatin filters (*p* = 0.0156 for both types of samplers), and similar findings were observed for the total bacterial concentrations (*p* = 0.004 and 0.012 for the Button and IOM samplers, respectively). However, the culturable fungal and fungal spore levels did not differ significantly among the three filter types. In total, 16 culturable bacterial species were observed among all the samples. Six, three, and four species were observed on the PC, PTFE, and gelatin filters, respectively, when coupled with the Button samplers. Four, four, and two species were observed on the PC, PTFE, and Gelatin filters, respectively, when coupled with the IOM samplers (Table 2). Slightly more species were recovered from the PC filters than from the other filters. Twelve culturable fungal taxa were observed among all the samples. The PC, PTFE, and gelatin filters coupled to the Button samplers recovered five, five, and six fugal taxa, and those coupled to the IOM samplers recovered four, four, and five taxa, respectively (Table 2). The gelatin filter recovered slightly more fungal taxa than the other filters.

**Table 2:** Relative efficiency comparison of filters.^a^

		Button sampler				IOM sampler			
		PC	PTFE	Gelatin	*p* value^*b*^	PC	PTFE	Gelatin	*p* value^*b*^
**Culturable bacteria (CFU/m** ^**3**^ **)** ^*c*^	Mean	1800	446	35	0.0070	1019	231	81	0.0119
	Median	833	208	0		1250	208	0	
	SD	2573	507	45		754	259	145	
**Overall number of culturable bacterial species recovered**		6	3	4		4	4	2	
**Culturable fungi (CFU/m** ^**3**^ **)**	Mean	145	162	104	0.7074	127	266	278	0.7275
	Median	52	52	104		0	0	104	
	SD	186	272	86		179	542	539	
**Overall number of culturable fungal taxa recovered**		5	5	6		4	4	5	
**Total bacteria (cells/m** ^**3**^ **)**	Mean	8.2×10^5^	5.0×10^5^	2.1×10^5^	0.0043	2.1×10^6^	1.0×10^6^	1.2×10^6^	0.0043
	Median	4.3×10^5^	2.2×10^5^	2.3×10^5^		6.6×10^5^	4.4×10^5^	5.8×10^5^	
	SD	8.8×10^5^	4.8×10^5^	1.1×10^5^		2.4×10^6^	1.2×10^6^	1.4×10^6^	
**Fungal spores (spore/m** ^**3**^ **)**	Mean	50	74	206	0.0794	93	167	167	0.1699
	Median	0	21	167		0	42	250	
	SD	69	99	216		141	280	157	

^*a*^Every combination of filter and sampler contained nine samples; the three tested filters included polycarbonate (PC) filters, polytetrafluoroethylene (PTFE) filters, and gelatin filters.

^*b*^Friedman test, α = 0.05.

^*c*^CFU/m^3^: colony forming units per cubic meter of air

Overall, the relative sampling efficiency of the PC filter was higher than that of the other two filters. Although the gelatin filter recovered slightly more fungal taxa than the other filters did, it dissolves in environments with high temperature and relative humidity, rendering it inappropriate for most occupational settings in Taiwan. Therefore, PC filters were used to further compare the performance of personal bioaerosol samplers.

### Relative efficiency evaluation of personal samplers

During the sampling periods, variations in the indoor environmental conditions were small, except the number of people (17 to 52). The temperature was 21.5 to 25.9°C; the relative humidity was 70.0% to 80.0%; and the wind speed was 0.03 to 0.13 m/s. By contrast, variations in the outdoor environmental conditions were large. The number of people ranged from 95 to 522; the temperature was 22.5 to 27.9°C; the relative humidity was 70.0% to 90.0%; and the wind speed was 0.18 to 0.45 m/s.

Table 3 lists a comparison of the performance of the four samplers in collecting culturable bacteria. The Button sampler recovered the highest concentrations of total culturable bacteria in the indoor and outdoor environments, whereas the Filter Cassette sampler recovered the lowest concentrations. Overall (*p* = 0.0043) and indoor (*p* = 0.0028) concentrations differed significantly among the four samplers; however, the outdoor concentrations (*p* = 0.1213) did not. According to the post hoc tests, significant differences among the overall bacterial concentrations were observed between the Button and Filter Cassette samplers (*p* = 0.0007), as well as between the IOM and Filter Cassette samplers (*p* = 0.0052). In total, 20 bacterial species were recovered from all the samples. More bacterial species were observed on the IOM, Button, and Filter Cassette samplers, with 16, 15, and 15 species, respectively. On average, the Button samplers recovered more bacterial species per plate (Table 3). Most of the bacterial taxa had the highest recovery frequencies in the Button and IOM samplers. Overall, *Staphylococcus cohnii* and *S*. *capitis* were the most prevalent bacterial species at both sampling locations. The overall recovery frequency (the percentage of samples with that specific bacterial species) of *S*. *cohnii* was 30.5% (40.0% in the Button sampler and 32.5% in the IOM sampler). The overall recovery frequency of *S*. *capitis* was 9.0% (12.5% in the Button sampler and 10.0% in the IOM sampler). These two species are the normal floras of the human skin and respiratory tract. Culturable bacterial floras were similar in the indoor and outdoor environments.

**Table 3:** Relative efficiency comparison of personal samplers based on culturable bacterial concentrations and diversity.^*a*^

		Button sampler	IOM sampler	Filter Cassette sampler	NIOSH sampler-Tube^*b*^	NIOSH sampler-Filter^*b*^	p value^*c*^
**Indoor concentrations (CFU/m** ^**3**^ **)** ^*d*^	Mean	4983	1523	255	185	3.91	0.0028
	Median	365	365	91	94	0	
	SD	20409	4087	588	320	17.47	
**Outdoor concentrations (CFU/m** ^**3**^ **)**	Mean	4261	2034	250	1675	16	0.1213
	Median	17	0	0	83	0	
	SD	18612	5770	774	3792	70	
**Overall concentrations (Indoor + Outdoor) (CFU/m** ^**3**^ **)**	Mean	4622	1779	252	940		0.0043
	Median	104	122	0	83		
	SD	19283	4942	678	2759		
**Overall number of bacterial species recovered**		15	16	15	12		—
**Average number of bacterial species recovered in each sample (range)**		1.3 (0–4)	1.1(0–4)	1 (0–4)	1.1 (0–4)		—

^*a*^Every combination contained 40 samples.

^*b*^The tube and filter samples of the NIOSH sampler were analyzed separately, but the concentrations were combined for the statistical analysis.

^*c*^Friedman test, α = 0.05.

^*d*^CFU/m^3^: colony forming units per cubic meter of air

The performance of the four samplers in collecting culturable fungi is summarized in Table 4. The four samplers showed significant differences. The Button and IOM samplers recovered higher overall concentrations of culturable fungi. According to the post hoc tests, significant differences existed between the Button and Filter Cassette samplers (*p* = 0.001), the Button and NIOSH samplers (*p* = 0.0012), the IOM and Filter Cassette samplers (*p* = 0.0003), as well as the IOM and NIOSH samplers (*p* = 0.0004). The relative collection efficiencies of the Button and IOM samplers were not significantly different. Similar trends were observed in the indoor and outdoor environments. In total, 36 fungal taxa were observed among all samples. The IOM, Button, and Filter Cassette samplers recovered 23, 20, and 19 fungal taxa, respectively, more than the NIOSH sampler. On average, the Button and IOM samplers recovered more fungal taxa per plate (Table 4). Most culturable fungi were recovered more frequently from the Button and IOM samplers. Overall, Non-sporulating fungi (the overall recovery efficiency 55.0%), Yeast (34.5%), *Penicillium* (26.0%), *Cladosporium* (23.0%), and *Aspergillus* (22.0%) were the most prevalent fungal taxa in the sampling locations. These fungi are commonly observed in indoor and outdoor environments and have been associated with allergic diseases and opportunistic infections [35–37]. Predominant fungal species were similar in the indoor and outdoor sampling locations, except that greater fungal variety was recovered outdoors (29 taxa) than indoors (18 taxa).

**Table 4:** Relative efficiency comparison of personal samplers based on total culturable fungal concentrations and diversity.^a^

		Button sampler	IOM sampler	Filter Cassette sampler	NIOSH sampler-Tube^*b*^		NIOSH sampler-Filter^*b*^	*p* value^*c*^
**Indoor concentrations (CFU/m** ^**3**^ **)** ^*d*^	Mean	1633	872	250	238	236		0.0049
	Median	313	339	104	115	0		
	SD	4806	1510	435	390	928		
**Outdoor concentrations (CFU/m** ^**3**^ **)**	Mean	1060	2195	904	515	22		< 0.0001
	Median	952	1879	801	458	0		
	SD	588	1680	670	525	36		
**Overall concentrations (Indoor + Outdoor) (CFU/m** ^**3**^ **)**	Mean	1347	1534	577	505			< 0.0001
	Median	833	653	305	229			
	SD	3392	1713	648	764			
**Overall number of fungal taxa recovered**		20	23	19	15			—
**Average number of fungal taxa recovered in each sample (range)**		2.9 (0–8)	2.6 (0–7)	2.2 (0–6)	2.1 (0–6)			—

^*a*^Every combination contained 40 samples.

^*b*^The tube and filter samples of the NIOSH sampler were analyzed separately, but the concentrations were combined for the statistical analysis.

^*c*^Friedman test, α = 0.05.

^*d*^CFU/m^3^: colony forming units per cubic meter of air

The performance of the four samplers in collecting total bacteria differed significantly (Table 5). The IOM sampler recovered higher total bacterial concentrations than the other samplers did in both indoor and outdoor environments. According to the post hoc tests, the overall concentrations differed significantly between the IOM and other samplers (*p* < 0.0001 for all contrasts), the Button and Filter Cassette samplers (*p* = 0.0026), as well as the NIOSH and Filter Cassette samplers (*p* = 0.0001).

**Table 5:** Relative efficiency comparison of personal samplers based on total bacterial concentrations.^a^

		Button sampler	IOM sampler	Filter Cassette sampler	NIOSH sampler-Tube^*b*^	NIOSH sampler-Filter^*b*^	*p* value^*c*^
**Indoor concentrations** (cells/m^3^)	Mean	5.7×10^5^	8.4×10^5^	5.3×10^5^	1.6×10^5^	3.7×10^5^	0.0001
	Median	4.1×10^5^	6.1×10^5^	2.7×10^5^	1.5×10^5^	2.3×10^5^	
	SD	4.7×10^5^	7.5×10^5^	8.3×10^5^	1.4×10^5^	3.5×10^5^	
**Outdoor concentrations** (cells/m^3^)	Mean	6.1×10^5^	1.1×10^6^	5.1×10^5^	3.7×10^5^	4.8×10^5^	< 0.0001
	Median	5.4×10^5^	1.0×10^6^	4.0×10^5^	2.9×10^5^	3.8×10^5^	
	SD	5.3×10^5^	9.0×10^6^	6.5×10^5^	5.9×10^5^	6.4×10^5^	
**Overall concentration (Indoor + Outdoor)** (cells/m^3^)	Mean	5.9×10^5^	9.7×10^5^	5.2×10^5^	6.9×10^5^		< 0.0001
	Median	5.0×10^5^	8.4×10^5^	3.8×10^5^	5.5×10^5^		
	SD	4.9×10^5^	8.3×10^5^	7.3×10^5^	9.3×10^5^		

^*a*^Every combination contained 40 samples.

^*b*^The tube and filter samples of the NIOSH sampler were analyzed separately, but the concentrations were combined for the statistical analysis.

^*c*^Friedman test, α = 0.05.

Table 6 lists a comparison of the performance of the four samplers in sampling fungal spores. The overall and outdoor concentrations differed significantly among the four samplers; however, the indoor concentrations did not. The highest overall fungal spore concentrations were observed in the IOM sampler, whereas the lowest concentrations were observed in the Filter Cassette sampler. According to the post hoc tests, the overall concentrations were significantly different between the IOM and the Filter Cassette samplers (*p* = 0.002), as well as the IOM and NIOSH samplers (*p* = 0.0041). In total, 24 fungal taxa were observed among all the samples. The NIOSH and Button samplers recovered greater fungal varieties compared with the other samplers, with 21 and 20 fungal taxa, respectively. These two samplers also recovered more fungal taxa per plate (Table 6). Most fungal taxa had the highest recovery frequencies in the Button sampler. Overall, Ascospores (the recovery frequency 56.0%), Basidiospores (56.0%), *Aspergillus/Penicilliun* (47.0%), and *Cladosporium* (44.5%) were the predominant fungal categories at the sampling sites. These fungal taxa are common in indoor and outdoor environments.

**Table 6:** Relative efficiency comparison of personal samplers based on total fungal spore concentrations and diversity.^a^

		Button sampler	IOM sampler	Filter Cassette sampler	NIOSH sampler-Tube^*b*^	NIOSH sampler-Filter^*b*^	*p* value^*c*^
**Indoor concentrations** (spores/m^3^)	Mean	161	153	116	82	21	0.1969
	Median	146	115	26	50	0	
	SD	143	158	169	92	40	
**Outdoor concentrations** (spores/m^3^)	Mean	1418	1848	786	1167	34	0.0139
	Median	807	1365	635	667	0	
	SD	1701	1491	916	1307	75	
**Overall concentration (Indoor + Outdoor)** (spores/m^3^)	Mean	789	1001	451	652		0.0064
	Median	313	453	156	283		
	SD	1351	1353	733	1069		
**Overall number of fungal taxa recovered**		20	16	18	21		—
**Average number of fungal taxa recovered in each sample (range)**		5.5 (0–15)	4.4 (0–11)	4.9 (0–15)	5.7 (0–14)		—

^*a*^Every combination contained 40 samples.

^*b*^The tube and filter samples of the NIOSH sampler were analyzed separately, but the concentrations were combined for the statistical analysis.

^*c*^Friedman fest, α = 0.05.

In summary, the Button and IOM samplers had the highest relative sampling efficiencies (i.e., higher recovered concentrations and biodiversity) in collecting bacteria and fungi, followed by the NIOSH sampler. Therefore, we further evaluated all the tested samplers, except the Filter Cassette samplers, in a pig farm to assess their performance in field sampling.

### Field evaluation

During the sampling periods for the field evaluation, the average temperature was 27±0.54°C (mean±SD), and the relative humidity was 72.9±1.19%. The concentrations of the other environmental factors, including the levels of suspended particulates, TVOC, CO, and CO_2_, were lower than the air quality standards and occupational exposure limits of Taiwan.

Table 7 presents the personal bioaerosol exposure data of the pig farm workers. For culturable fungi, the IOM sampler recovered the highest concentrations. For fungal spores, the Button and IOM samplers recovered higher concentrations. For culturable and total bacteria, no obvious differences were observed between the concentrations recovered by the three samplers. A comparison of the cleaning methods showed that most bioaerosol concentrations were substantially higher when the workers used the dry method to clean sheds (sweeping sheds in the morning) in contrast with the wet method (washing sheds in the afternoon).

**Table 7:** Concentrations of personal bioaerosol exposure in a pig farm.

	Sampling period^*a*^	Button sampler	IOM sampler	NIOSH sampler^*b*^
**Culturable bacteria (CFU/m** ^**3**^ **)**	Morning (feeding + dry cleaning)	28401	17714	29553
	Afternoon (feeding + wet cleaning)	495	2217	6734
	Full day	43438	54553	9053
**Culturable fungi (CFU/m** ^**3**^ **)** ^*c*^	Morning (feeding + dry cleaning)	1988	3769	834
	Afternoon (feeding + wet cleaning)	371	985	693
	Full day	2439	2871	1471
**Total bacteria (cells/m** ^**3**^ **)**	Morning (feeding + dry cleaning)	4.9×10^6^	6.2×10^6^	1.1×10^6^
	Afternoon (feeding + wet cleaning)	2.9×10^6^	5.3×10^5^	4.0×10^6^
	Full day	3.7×10^6^	6.4×10^6^	1.0×10^7^
**Fungal spores (spores/m** ^**3**^ **)**	Morning (feeding + dry cleaning)	4317	2827	4281
	Afternoon (feeding + wet cleaning)	3885	2069	1659
	Full day	6874	7379	2461

^*a*^n = 1 for each sampling period; the samples of Morning, Afternoon, and Full Day are three independent samples and Full day data did not relate to the average data of Morning + Afternoon.

^*b*^Bioaerosol concentrations of the NIOSH sampler included results from the main tube and backup filter.

^*c*^CFU: colony forming unit

The main culturable bacterial species recovered were the normal floras of human and pig skin and respiratory tracts, such as *S*. *capitis* and *S*. *cohnii*. Because the pig farm was a half-open environment, the most prevalent fungi were common outdoor taxa, including Ascospores, Basidiospores, *Cladosporium*, and Non-sporulating fungi.

## Discussion

### Filter Comparison

To select the optimal sampling medium for the tested filter samplers, we first compared the relative collection efficiencies of three filter types (gelatins, PTFE, and PC filters) based on recovered bioaerosol concentrations and biodiversity. According to our results, no significant differences existed among the three filter types in sampling culturable fungi and fungal spores. However, for the culturable and total bacteria, the PC filter collected significantly higher concentrations than the other two filters (Table 2). Our findings were inconsistent with those obtained by Burton et al. [31]. In their study, the objective was to determine filter materials (MCE, PTFE, gelatin, and PC) and extraction methods appropriate for the environmental sampling of *Bacillus subtilis* endospores. The authors found that 1-μm PTFE and gelatin filters had similar average physical collection efficiencies of 94% or higher. The 3-μm PC filters had a lower collection efficiency of 61%. The discrepancy is probably because the pore size of their tested PC filters (3μm) was considerably larger than ours (0.8μm). Subsequently, Burton et al. [15] compared three filter types (PTFE, gelatin, and PC) to determine the physical collection efficiency for *Bacillus atrophaeus* endospores (0.9μm). Our results were consistent with this study. The authors found that the 1-μm PC filters had a higher physical collection efficiency than the 3-μm gelatin and 1-μm PTFE filters. Van Droogenbroeck et al. [22] used the IOM sampler loaded with four types of filters (MCE, glass fiber, gelatin, and PC) to determine the collection efficiency for *Chlamydophila psittaci*. They found that the 3-μm gelatin filters had a higher collection efficiency than the 0.1-μm PC filter. However, the pore size of the tested PC filters was smaller than ours (0.8μm), and the authors used a smaller flow rate (1 L/min) to avoid filter blocking and distortion. Both of these factors may influence the collection efficiency of PC filters.

Considering the physical properties of the filters, the PTFE and gelatin filters are multi-layered filters that can use the gaps between the layers to catch particles with a diameter smaller than their pore sizes. The depth of the PC filter is smaller than that of the other two filters. The PC filter can retain particles on its smooth surface, which is ideal for microscopic analysis [38,39]. The gelatin filter (SKC Inc.) is composed of gelatin, containing 46% to 49% water, with good solubility [39]. Therefore, during sampling, the gelatin filter can retain higher microorganism viability than the other membrane filters can (i.e., PC and PTFE filters). However, the influence of environmental temperature and relative humidity on the gelatin filter after prolonged sampling should be considered.

According to the SKC operating instructions, the gelatin filter can tolerate a maximum room temperature of 30°C and a relative humidity of 85% [39]. The maximum recommended sampling time is 30 min, with a maximum recommended air face velocity of 0.4 m/s. Lower air velocities allow for longer sampling times. A prolonged sampling time, high air velocity, as well as high temperature and humidity may destroy the gelatin pores, thus leading to a decreased collection efficiency of the gelatin filters. During our filter evaluation, the highest temperature and relative humidity were 22.9°C and 72.5%, respectively, and the air velocities of the two filter samplers were lower than 0.4 m/s (IOM sampler = 0.08 m/s, Button sampler = 0.16 m/s). However, we were uncertain as to how long the sampling time could be extended under our study conditions. Moreover, we found that after 2 and 4 h of air sampling, the gelatin filters became fragile and slightly dissolved. This observation was consistent with that of Burton et al. [31]. Even under controlled temperature and humidity conditions, the gelatin filters were still brittle and broke easily after 4 h of air sampling. Once the filter pores are deformed, smaller bioaerosols can easily pass through the filters with the airflow, resulting in lower detected concentrations, while larger bioaerosols may still remain on the filters.

Although the PTFE filter is multi-layered, identical to the gelatin filter, it is highly hydrophobic and cannot sustain bioaerosol viability. In addition, completely extracting bioaerosols from the PTFE filter was difficult, thus resulting in a low relative collection efficiency in our study. Our extraction method was based on the results obtained by Wang et al. [16], who obtained an extraction efficiency of 96% to 98% even for the most sensitive microorganisms; however, their filter was a PC filter, and the efficiency of the extraction method performed using a PTFE filter may not be as high.

In summary, the PC filter exhibited the optimal relative collection efficiency for bacteria, which is a likely result of its smaller pore size (0.8 μm) and physical characteristics. By contrast, the gelatin filter exhibited a superior performance in collecting fungal spores though the result was not statistically significant. This may be explained by the ability of the pores (3μm in diameter) to retain the relatively large fungal spores despite being distorted by prolonged sampling and adverse environmental conditions.

### Sampler comparison

The culturable bacterial and fungal concentrations recovered in this study were in similar ranges as other studies conducted in Northern Taiwan. The use of culture-based impactors resulted in the average culturable fungal and bacterial concentrations of 427–1444 CFU/m^3^ and 282–812 CFU/m^3^, respectively, in five long-term care facilities in Taipei [40]; total culturable bacterial levels of 931 CFU/m^3^ and 869 CFU/m^3^ in the lobbies of two hospitals [41]; and a mean total culturable bacterial level of 698 CFU/m^3^ in a hospital [42]. A study conducted in traditional markets in Southern Taiwan revealed that the culturable bacterial and fungal concentrations were approximately 4500 CFU/m^3^ and 5000 CFU/m^3^, respectively [43]. The total bacterial and fungal spore counts are rarely studied in hospitals and traditional markets.

According to our results, the Button and IOM samplers had the optimal relative collection efficiencies for bacteria and fungi, followed by the NIOSH sampler. This result is consistent with those of a study conducted by Zamengo et al. [3], according to which, the collection efficiencies of the Button and IOM samplers did not differ. However, the optimal homogeneity of particle deposition was observed in the Button sampler. Moreover, Lee et al. [44] found that the Button and IOM samplers did not differ in their sampling efficiencies for inhalable particles in the wood products industries. Similar results were observed by Gorner et al. [45], who found that the Button and IOM samplers, when tested in a modeled wind tunnel, had a similar effect under calm air (<0.3 m/s) conditions. Even under moving air (1 m/s), the sampling efficiency of the Button sampler was only slightly lower than that of the IOM sampler. The authors also indicated that a 37-mm Filter Cassette sampler had the lowest sampling efficiency.

Regarding the biodiversity of fungal spores, our results indicated that the NIOSH and Button samplers recovered a greater variety of fungal spores. The total number of fungal taxa recovered and the average number of fungal taxa per plate were similar for both types of samplers (Table 6). This finding was similar to the results obtained by Macher et al. [25], who compared the sampling efficiencies of the NIOSH sampler and three other common samplers for collecting fungal spores. Their results also showed that the NIOSH sampler had a superior collection efficiency.

Wang et al. [16] found that the sampling efficiencies of the Button and 37-mm Filter Cassette samplers did not differ significantly for fungi and bacteria, except under high relative humidity conditions. At 85% relative humidity, the Button sampler had a significantly higher sampling efficiency for bacteria (*B*. *subtilis*) compared with the Filter Cassette sampler. In our study, this may partly explain why the relative sampling efficiency of the 37-mm Filter Cassette sampler was lower than that of the Button sampler. The relative humidity of the indoor/outdoor environments was high (70%–90%) when we compared the efficiencies of the personal samplers. Under this condition, more moisture collected on the filters of the Button sampler, resulting in a higher sampling efficiency.

Consistent with many of the aforementioned studies, our results indicated that the Button and IOM samplers had the optimal sampling efficiencies and that the Filter Cassette sampler had the lowest sampling efficiency among the three filter-based samplers. In addition to relative humidity, the air face velocity (0.16 m/s, 0.08 m/s, and 0.07 m/s for the Button, IOM, and 37-mm Filter Cassette samplers, respectively) may be another major factor contributing to the difference in collection efficiencies; a higher air face velocity results in an improved sampling efficiency.

The lower relative collection efficiency of the NIOSH sampler may partly be due to its focus on respirable dust sampling (d_50_ = 1.5 μm) compared with that of the Button and IOM samplers, which focus on inhalable dust sampling (d_50_ = 100 μm). The NIOSH sampler does offer a size-selective separation of various bioaerosol ingredients, which can be a unique feature if preferred by users. According to our results, the NIOSH sampler had a high relative collection efficiency for total bacteria, which is comparable with that of the Button sampler. However, the collection efficiency was inconsistent for culturable bacteria. This is probably due to the sampling principle and medium. The NIOSH sampler is a centrifugal sampler and uses inertial impaction to separate heavier particles from the air stream and deposit them at the bottom of the centrifuge tube. The residual smaller particles are then collected onto the backup filter. In this study, we did not coat any materials or add any collection liquid to the centrifuge tubes, which may have resulted in the death of bioaerosols in the process of sampling impaction. Although Chen et al. [26] reported that coating the surface of centrifuge tubes with polyethylene glycol was not beneficial for improving the collection efficiency, they found that adding water to the tubes resulted in a slight increase in collection efficiency. Therefore, in future studies, researchers may consider adding collection liquid to the centrifuge tubes to improve the culturable efficiency of the NIOSH sampler, especially for long-term sampling. In addition, a separate analysis of the tube and filter of the NIOSH sampler was conducted in this study to examine whether the filter should be analyzed. The results (shown in Tables 3, 4, 5, and 6) indicated that the filter contributed significantly; hence, it should be included in the analysis.

The concentrations of total culturable fungi and bacteria obtained by our personal exposure assessment of the pig farm workers were similar to those obtained in another study conducted in Taiwan. The authors found that the mean culturable fungal and bacterial levels were 3833 CFU/m^3^ and 38,903 CFU/m^3^, respectively, in six pig farms, using a filter-based method [46].The total fugal spore concentration in this study was slightly lower than that in another study conducted in a pig farm in Taiwan (3.2 × 10^5^ spores/m^3^) [47]. The total bacterial concentrations in this study were in the range of 10^5^–10^7^ cells/m^3^, similar to those obtained in the farms for laying hens (1.1–9.6 × 10^7^ cells/m^3^) [48] and to the geometric mean obtained for grain handling (4 × 10^6^ cells/m^3^) [49]. The most prevalent culturable fungus observed in this study was *Cladosporium*, consistent with the result of a previous study conducted in pig farms in Taiwan [46]. *Cladosporium* is a crucial outdoor allergen [35,37]; thus, its potential health risk in pig farms should be noted. Another problem that we encountered in the field evaluation of the samplers involved filter clogging. Although the collection efficiencies of the Button and IOM samplers were similar for culturable bacteria and fungi, as well as for fungal spores, the Button sampler was clogged during the 8-h outdoor sampling and the personal exposure assessment of field evaluation because of the higher airborne dust concentrations as well as the higher flow rate of the sampler (4 L/min). Therefore, the IOM sampler, with its lower flow rate (2 L/min), may be a more appropriate choice for workplaces with high dust levels.

### Effects of other factors on sampling efficiencies

The length of the sampling time and the properties of sampling media influence the collection efficiencies of samplers. In addition to the centrifuge tube mentioned previously, filters may also reduce the viability of bioaerosols. Because the sampling principle of filters is filtration, once bioaerosols are trapped, they must undergo desiccation before the sampling ends, which possibly causes the loss of viability [50,51]. Durand et al. [52] investigated the effect of sampling time on the viability of airborne fungi and bacteria. They used the 37-mm Filter Cassette sampler (2 L/min), along with the 0.8-μm PC filters for sampling at six composting facilities with high bioaerosol exposure. The results showed that even if the sampling period was prolonged to 6 h, it still did not influence the viability of bioaerosols normally present at composting facilities. The authors also reported that using PC filters for personal exposure assessment for a full work shift was a practical method in environments such as composting facilities. However, the authors did not compare the collection efficiencies of the filter samplers with those of the Andersen sampler or an impinger, which were found to have higher efficiencies than filter methods in a previous study [53].

According to the foregoing discussion, the use of PC filters for long-term sampling in our test environments with high bioaerosol concentrations and relative humidity seemed to exert a minimal impact on the viability of bioaerosols. However, the properties of filters and the length of the sampling time must still be determined carefully based on the study purpose, sampling locations, and target bioaerosols.

A limitation of our study is that we conducted the sampling in fixed locations to compare the efficiencies of the filters and personal samplers. The observed concentrations may be different from the results of real personal sampling. However, this should not influence the relative collection efficiencies of the filters and samplers. Many studies have used similar sampling methods to evaluate the collection efficiencies of personal samplers for bioaerosols [3,20,25]. Another limitation is that we did not control the environmental conditions and bioaerosol levels while comparing the sampling efficiencies; hence, we could not estimate the difference between the observed and actual bioaerosol concentrations. The observed concentrations of the tested filters and samplers were compared to evaluate the overall relative efficiencies. To improve the reliability of our results, we performed sampling for various time periods and increased the number of replicates. Similar methods have been applied before [20].

## Conclusion

In this study, we compared the overall collection/analysis efficiencies of three filters and four personal samplers for multiple bioaerosols. The PC filters had the highest relative efficiency among the three tested filters, particularly for the total concentrations and the diversity of bacteria. The Button and IOM samplers had higher overall collection/analysis efficiencies, followed by the NIOSH sampler. However, the Button sampler is more likely to be clogged in high airborne dust concentrations due to its higher flow rate (4 L/min), whereas the NIOSH sampler requires liquid in the tube for sustaining the culturability of the microbes for long-term sampling. Therefore, to conduct personal sampling in environments with high dust levels, the IOM sampler is a more appropriate choice. In summary, the Button and IOM samplers with PC filters (pore size 0.8 μm) are efficient collection methods for the personal exposure assessment of multiple bioaerosols. Nevertheless, the airborne dust level in the sampling environment should be considered when selecting the optimal sampler. In this study, we focused on the overall efficiency and the field application of the sampling/analysis methods. Future studies can further differentiate the various aspects of sampler efficiencies (i.e., physical, biological, and assay efficiencies).
